# Acetylation-mediated degradation of HSD17B4 regulates the progression of prostate cancer

**DOI:** 10.18632/aging.103530

**Published:** 2020-07-17

**Authors:** Huichao Huang, Ruijie Liu, Yahui Huang, Yilu Feng, Ying Fu, Lin Chen, Zhuchu Chen, Yi Cai, Ye Zhang, Yongheng Chen

**Affiliations:** 1 Department of Oncology, NHC Key Laboratory of Cancer Proteomics, XiangYa Hospital, Central South University, Changsha 410008, China; 2 Department of Pathology, XiangYa Hospital, Central South University, Changsha 410008, China; 3 Department of Pathology, XuChang Central Hospital, XuChang 461670, China; 4 Molecular and Computational Biology Program, Departments of Biological Sciences and Chemistry, University of Southern California, Los Angeles, CA 90089, USA; 5 Department of Urology, XiangYa Hospital, Central South University, Changsha 410008, China; 6 National Clinical Research Center for Geriatric Disorders, XiangYa Hospital, Central South University, Changsha 410008, China

**Keywords:** prostate cancer, 17β-Hydroxysteroid dehydrogenase type 4 (HSD17B4), acetylation, steroidogenic enzymes, chaperone-mediated autophagy

## Abstract

Steroidogenic enzymes are crucial in prostate cancer (PCa) progression. 17β-Hydroxysteroid dehydrogenase type 4 (HSD17B4), encoded by HSD17B4, lacks catalytic capacity in androgen metabolism. Now the detailed role and molecular mechanism of PCa development are largely unknown. Here we showed that the expression of HSD17B4 was increased in PCa tissues compared to paired paratumor tissues. HSD17B4 knockdown in PCa cells significantly suppressed its proliferation, migration and invasion, while overexpressing HSD17B4 had opposite effects. Mechanistically, we found that the protein level of HSD17B4 was regulated by its acetylation at lysine 669(K669). Dihydroxytestosterone (DHT) treatment increased HSD17B4 acetylation and then promoted its degradation via chaperone-mediated autophagy (CMA). SIRT3 directly interacted with HSD17B4 to inhibit its acetylation and enhance its stability. In addition, we identified CREBBP as a regulator of the K669 acetylation and degradation of HSD17B4, affecting PC cell proliferation, migration and invasion. Notably, in PCa tissues and paired paratumor tissues, the level of HSD17B4 was negatively correlated with its K669 acetylation. Taken together, this study identified a novel role of HSD17B4 in PCa progression and suggested that HSD17B4 and its upstream regulators may be potential therapeutic targets for PCa intervention.

## INTRODUCTION

Prostate cancer (PCa) is one of the most common and lethal malignant tumors in men worldwide [1]. Steroidogenic enzymes are essential biomarkers for disease diagnosis or potential therapy targets for PCa treatment [2].

Steroidogenic enzymes that accelerate potent androgen synthesis have been studied in-depth as they relate to driving PCa progression and treatment resistance [3]. CYP17A is the rate-limiting enzyme for cholesterol to dehydroepiandrosterone (DHEA) metabolism, providing androgen precursor for PCa. Abiraterone, an inhibitor of CYP17A, is used for castration-resistant PCa (CRPC) treatment [4]. 3βHSD1 converts DHEA to dihydrotestosterone (DHT). One SNP in 3βHSD1 prolongs its protein half-life and leads to a poorer response to clinical therapy [5]. Ectopic expression of HSD17B5 has been reported as a mechanism of drug resistance [6]. Thus, steroidogenic enzymes promoting active androgen synthesis play oncogenic roles in PCa development.

However, steroidogenic enzymes involved in inactivating androgen have not been thoroughly exploited thus far. Isoform 2 of the HSD17B4 enzyme catalyzes the reverse reaction of HSD17B5 [7]. It catalyzes 17β-OH to 17-keto, resulting in androgen inactivation [8]. However, the full-length HSD17B4 has lost its enzyme activity in androgen metabolism [7]. The function and regulatory mechanism of HSD17B4 in PCa are elusive.

HSD17B4 is overexpressed in various cancers, including breast cancer, hepatocellular carcinoma, colonic cancer and ovarian cancer [9–12]. Elevated HSD17B4 levels have been correlated with poor prognosis and resistance to chemotherapy and radiation therapy in many solid tumors. However, little is known about the role of HSD17B4 in PCa. Although some studies demonstrated that HSD17B4 is involved in PCa progression, and the regulation of HSD17B4 expression has been studied at the transcriptional level, its posttranslational regulation in PCa remains unknown.

Covalent lysine acetylation has been identified as an evolutionarily conserved modification in metabolic enzymes and plays critical roles in the regulation of multiple enzymes [13–16]. In this study, we demonstrate that HSD17B4 is acetylated at lysine residue 669 in response to DHT and that K669 acetylation promoted the degradation of HSD17B4 via CMA [4, 10]. Explaining the perplexing link between HSD17B4 acetylation and the development of PCa can provide new insights into disease surveillance, therapeutic control, and, ultimately, an efficient treatment for advanced PCa.

## RESULTS

### The expression of HSD17B4 is increased in PCa development

**Figure 1:**
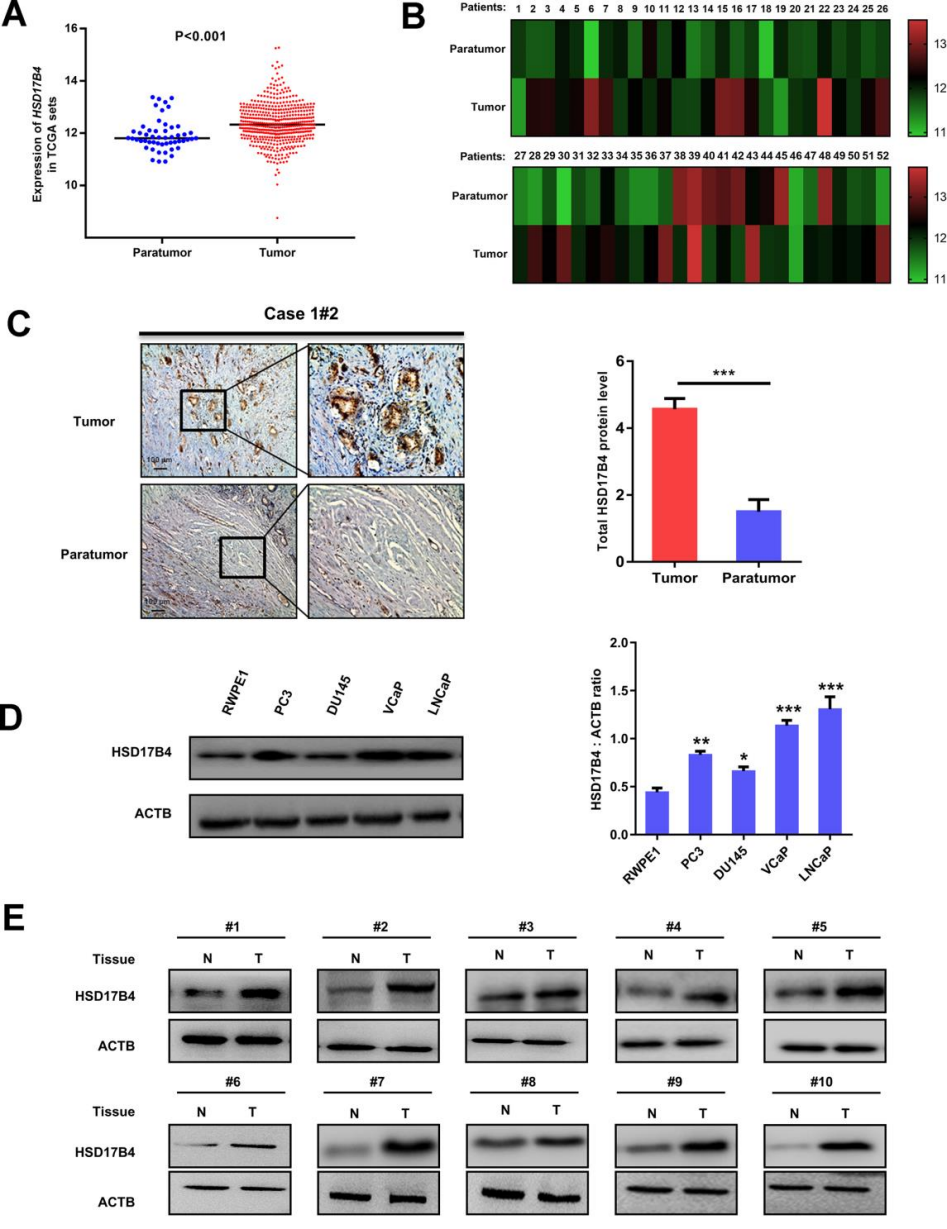
**Expression of HSD17B4 is increased as PCa develops.** (**A**) The expression of HSD17B4 was frequently upregulated in 498 PCa tissues (Tumor) compared with 52 adjacent normal prostate tissue samples (Paratumor) in the TCGA profile. (**B**) HSD17B4 expression was markedly increased in 52 paired PCa tissues (Tumor) and their adjacent normal tissues (Paratumor) in the TCGA profile. (**C**) Immunohistochemical staining of total HSD17B4 protein in tumor and adjacent tissues. A total of 10 PCa tissues and 10 adjacent normal prostate tissues were analyzed. Quantitative analysis of total HSD17B4 expression was performed by ImageJ. (**D**) Characterization of total HSD17B4 protein levels in PCa cell lines. Equal amounts of protein (20 μg) were immunoblotted for HSD17B4 and ACTB (loading control), as shown in the left panel. The intensities of total HSD17B4 protein are quantified in the right panel. (**E**) HSD17B4 is overexpressed in PCa tissues compared to expression in adjacent tissues. Human PCa samples each paired with cancerous tissue (designated as T) and adjacent normal tissue (designated as N) were lysed and directly subjected to western blotting. Ten pairs of samples clearly exhibited HSD17B4 overexpression in PCa tissues. Data are shown as the mean ± SD (n = 3) or typical photographs of one representative experiment. Similar results were obtained in three independent experiments. *p < 0.05, **p < 0.01, ***p <0.001.

Previous results have reported that HSD17B4 is a multiple-function enzyme involved in the progression of various cancers, including PCa [9, 17]. To elucidate the role of HSD17B4 in PCa pathogenesis, we analyzed *HSD17B4* mRNA levels in published profiles from PCa patients and found that HSD17B4 was upregulated in PCa samples (498 cases) compared with levels in adjacent normal tissue samples (52 cases) (P < 0.001) (Figure 1A and Supplementary Table 2). Furthermore, we found that the expression of HSD17B4 in a total of 52 paired PCa tissues was significantly higher compared with that in their adjacent normal tissues (P < 0.01) by TCGA dataset analysis (Figure 1B). Then, we examined the HSD17B4 protein level in human PCa tissues. We collected 10 pairs of paraffin-embedded human PCa samples with adjacent normal tissues for immunohistochemistry. The results showed that, compared with the matched normal prostate tissues, 10 pairs of samples showed a significant increase (P < 0.001) in total HSD17B4 protein in cancerous tissues (Figure 1C and Supplementary Table 1). Then, we collected several PCa cells (LNCaP, VCaP, DU145, and PC3) and a nonmalignant human prostate epithelial cell line (RWPE-1). HSD17B4 protein levels were examined by western blotting. The results showed that the level of HSD17B4 was higher in malignant PCa cells than that in normal cells (Figure 1D, left panel), and statistical analysis revealed the difference in HSD17B4 protein expression in those prostate cells (P < 0.001) (Figure 1D, right panel). Furthermore, we collected 20 pairs of human PCa tissues with adjacent normal prostate tissues for direct immunoblotting. In most samples, the levels of total HSD17B4 protein were higher in the tumor tissues compared with levels in the normal prostate tissues (Figure 1E and Supplementary Figure 1). Taken together, these data imply an important role of HSD17B4 in PCa and indicate a prospective application of using HSD17B4 as a potential biomarker for PCa diagnosis.

### HSD17B4 enhances malignant phenotypes in PCa cells

**Figure 2:**
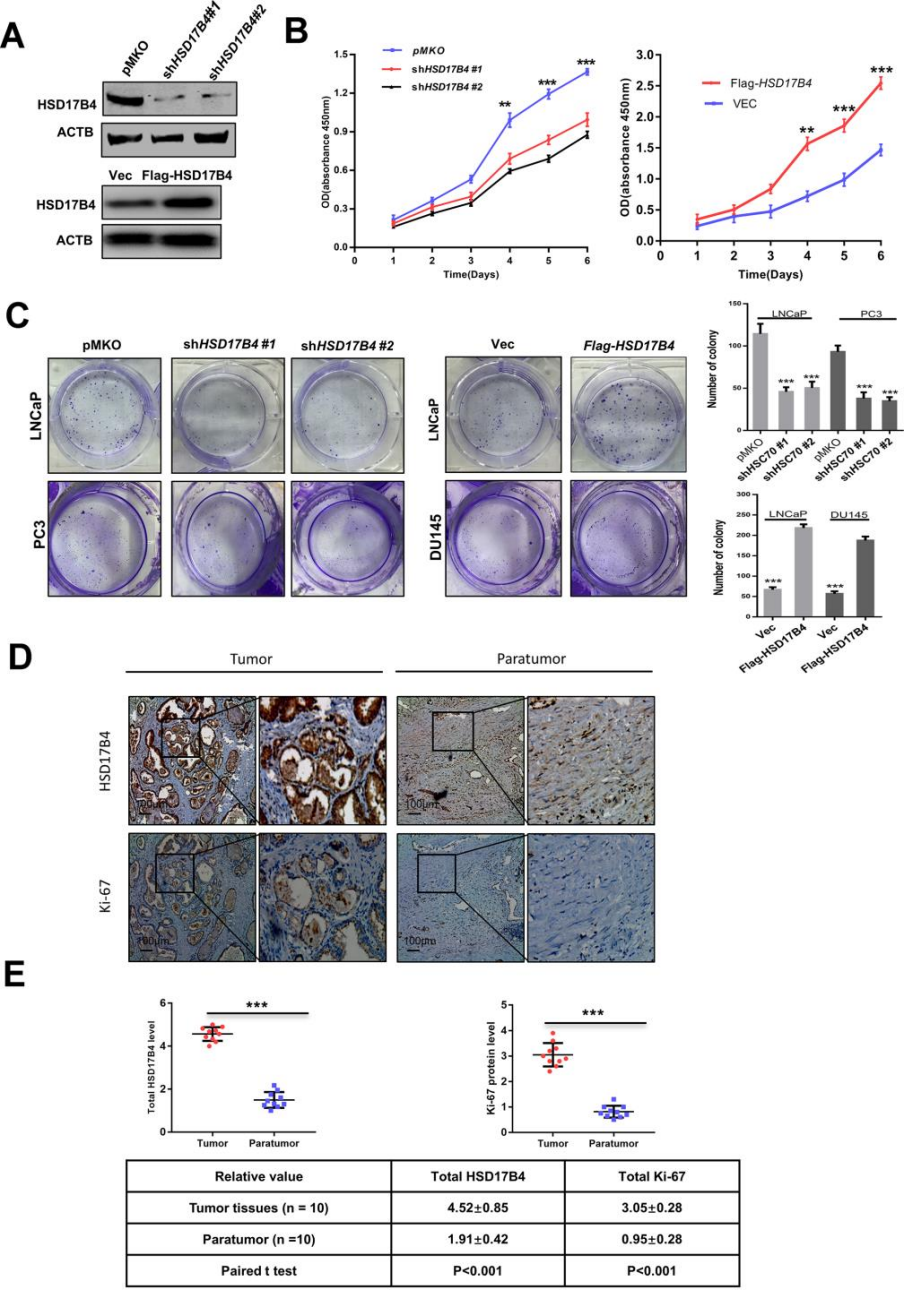
**HSD17B4 promotes the proliferation of PCa cells.** (**A**) Verification of LNCaP stable cell lines. The transfection efficiencies of sh*HSD17B4* and Flag-HSD17B4 were determined by western blotting. (**B**) *HSD17B4* knockdown inhibited LNCaP cell growth, while *HSD17B4* overexpression promoted cell growth. The CCK-8 assay showed that the growth of LNCaP cells characterized in (**A**) was affected by *HSD17B4* knockdown or overexpression. The data shown are representative of three independent experiments. (**C**) HSD17B4 promotes the proliferation of PCa cells. PCa cells were transfected with sh*HSD17B4* or Flag-HSD17B4 plasmids as indicated and analyzed by a colony formation assay (left panel). Quantitative analysis of the colony was performed by ImageJ. **denotes P < 0.01; ***denotes P< 0.001. Error bars represent ±SD of triplicate experiments (right panel). (**D**–**E**) Immunohistochemical staining of Ki-67 and total HSD17B4 protein in tumor and adjacent tissues. Examples are shown in (**D**), and the statistical analysis of all samples is shown in (**E**). Scale bars: 100 μm. The intensities of the HSD17B4 and Ki-67 proteins in 10 PCa tissues (upper panel) and 10 adjacent normal prostate tissues (lower panel) were quantified, followed by statistical analysis. The mean value of multiple samples and the standard deviation are presented. Data are shown as the mean ± SD (n = 3) or typical photographs of one representative experiment. Similar results were obtained in three independent experiments. *p < 0.05, **p < 0.01, ***p <0.001.

To explore the function of HSD17B4 in cancer cells, we knocked down endogenous *HSD17B4* by shRNA or overexpressed exogenous Flag-HSD17B4 in PCa cells (Figure 2A and Supplementary Figure 2A) and detected a significant difference in the proliferating velocity and colony formation (Figure 2B–2C and Supplementary Figure 2B). Ki-67 is a nuclear nonhistone protein that is universally expressed among proliferating cells and absent in quiescent cells [18, 19]. Research studies have shown that high levels of Ki-67 are associated with poor malignant cancer survival [20, 21]. The finding that HSD17B4 can increase PCa cell proliferation prompted us to examine both HSD17B4 and Ki-67 protein in human PCa tissues. We collected a total of 10 primary human PCa samples and 10 adjacent normal prostate samples and determined the levels of both HSD17B4 and Ki-67 protein by immunohistochemistry in paraffin-embedded tissues. In most samples, the levels of both HSD17B4 and Ki-67 protein were higher in the tumor tissues compared with levels in the normal prostate tissues (Figure 2D). Statistical analysis of quantified images indicated that the differences in HSD17B4 protein levels (P < 0.001) and in Ki-67 protein levels (P < 0.001) between tumor and normal tissues are all significant (Figure 2E). These data indicate that HSD17B4 may be a potential biomarker together with Ki-67 in predicting proliferative PCa.

**Figure 3:**
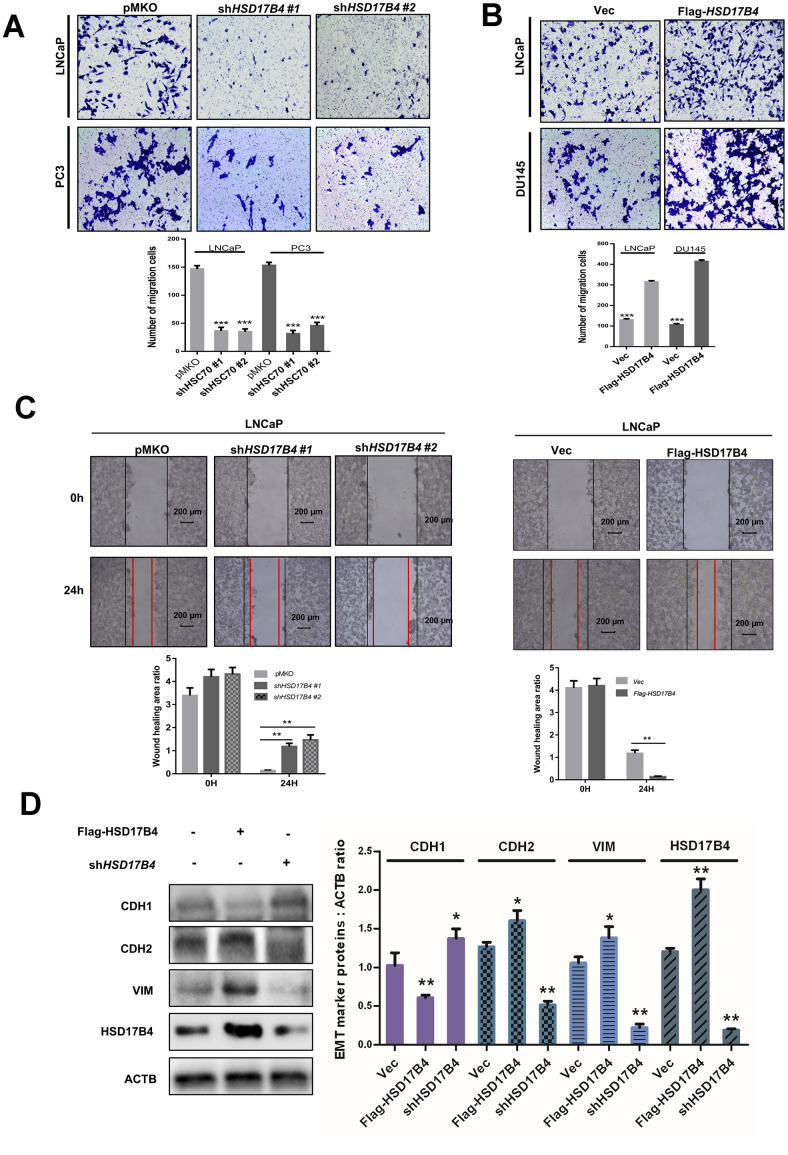
**HSD17B4 promotes the migration of PCa cells.** (**A**–**B**) Knockdown of *HSD17B4* inhibits the migration of PCa cells, while *HSD17B4* overexpression promotes cell migration. LNCaP, DU145 and PC3 stable cells were treated as indicated and analyzed by migration assays in 24-well chambers without Matrigel. Quantitative analysis of cell migration was performed by ImageJ. (**C**) Knockdown of *HSD17B4* inhibits migration of PCa cells, while *HSD17B4* overexpression promoted cell migration. LNCaP, DU145 and PC3 stable cells treated as indicated were analyzed by a wound-healing assay. Scale bars: 200 μm. Quantitative analysis of the wound healing area was performed by ImageJ. (**D**) Knockdown of *HSD17B4* increases CDH1 but decreases CDH2 and VIM in LNCaP cells, while *HSD17B4* overexpression induces the inverse results (left panel). Protein levels of CDH1, CDH2, VIM and HSD17B4 were normalized against ACTB, *denotes P<0.05, ^**^denotes P < 0.01. Error bars represent ±SD of triplicate experiments (right panel). Data are shown as the mean ± SD (n = 3) or typical photographs of one representative experiment. Similar results were obtained in three independent experiments. *p < 0.05, **p < 0.01, ***p <0.001.

Next, we performed migration assays in 24-well chambers and found that HSD17B4 knockdown significantly suppressed the migration of PCa cells, while overexpression of HSD17B4 promoted the number of migrating cells (Figure 3A–3B). The wound-healing assay obtained a similar result in which HSD17B4 regulated the migration of PCa cells (Figure 3C). EMT means that cells transit from an epithelial phenotype to mesenchymal properties. CDH1 is decreasing while CDH2 and Vimentin are increasing during EMT, and then cells lose their polarity and adhesiveness and subsequently migrate faster. Interestingly, we knocked down *HSD17B4* in LNCaP cells and found an increase in CDH1 but a decrease in CDH2 and Vimentin. However, the overexpression of HSD17B4 plays an opposite role (Figure 3D). These findings demonstrate that HSD17B4 plays important roles in enhancing the malignant capacities of PCa cells.

### K669 acetylation of HSD17B4 promotes its degradation in PCa cells

**Figure 4:**
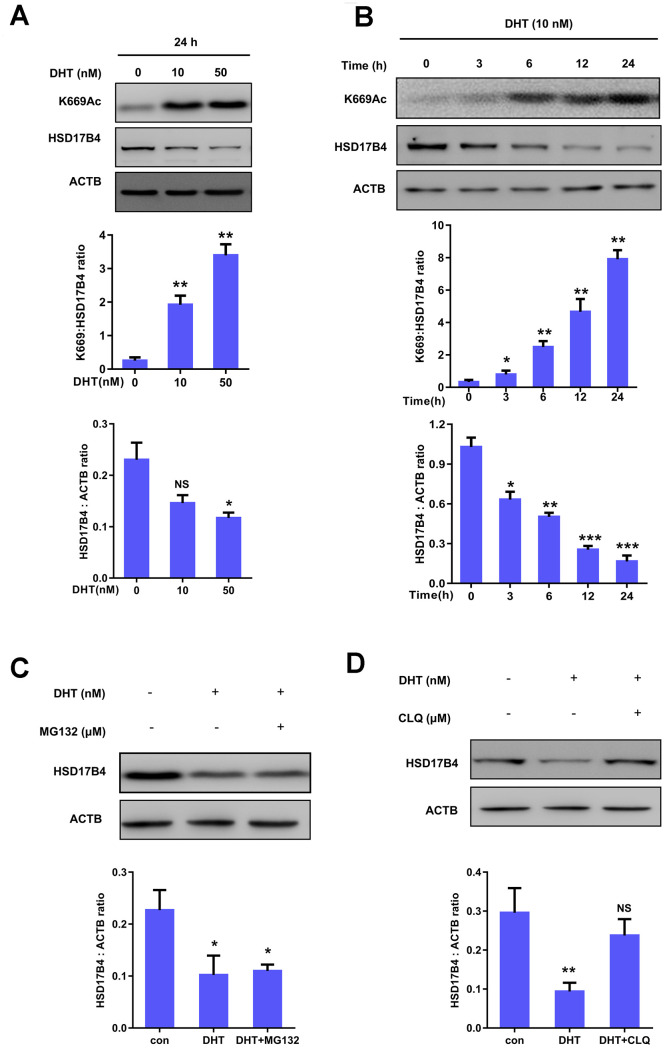
**K669 acetylation of HSD17B4 promotes its degradation in PCa cells.** (**A**) DHT treatment increases HSD17B4 K669 acetylation levels but decreases endogenous HSD17B4 protein levels in a dose-dependent manner. LNCaP cells were either untreated or treated with DHT at different concentrations as indicated. The K669 acetylation level and the protein steady-state level of HSD17B4 were determined by western blotting and normalized against ACTB (upper panel). The relative K669 protein level compared with the HSD17B4 level (middle panel) and relative HSD17B4 protein level compared with the ACTB level (lower panel) were quantified. ^*^denotes P < 0.05, **denotes P<0.01. Error bars represent ±SD for triplicate experiments. (**B**) DHT treatment increases HSD17B4 K669 acetylation while decreasing its protein in a time-dependent manner. The HSD17B4 acetylation level and protein level were determined by western blotting after treatment with DHT for different lengths of time, as indicated in LNCaP cells (upper panel). The relative K669 protein level compared with the HSD17B4 level (middle panel) and relative HSD17B4 protein compared with the ACTB level (lower panel) were quantified. ^*^denotes P < 0.05, ^**^denotes P < 0.01, ***denotes P<0.001. Error bars represent ±SD for triplicate experiments. (**C**) HSD17B4 is not degraded by the ubiquitin proteasome system (UPS). LNCaP cells were treated as indicated, and HSD17B4 protein levels were analyzed by western blotting (upper panel). Relative HSD17B4 protein compared with the ACTB level was quantified. ^*^denotes P < 0.05. Error bars represent ±SD for triplicate experiments (lower panel). (**D**) The lysosome inhibitor chloroquine (CLQ) restores HSD17B4 protein that was reduced by DHT treatment. LNCaP cells were treated with the lysosome inhibitor CLQ, and the HSD17B4 protein level was analyzed by western blotting (upper panel). The relative HSD17B4 protein compared with the ACTB level was quantified. ^**^denotes P < 0.01, NS, no significance. Error bars represent ±SD for triplicate experiments (lower panel). Data are shown as the mean ± SD (n = 3) or typical photographs of one representative experiment. Similar results were obtained in three independent experiments. *p < 0.05, **p < 0.01, ***p <0.001.

We further investigated the molecular mechanism of HSD17B4 in PCa. To determine whether DHT can serve as a physiological signal and dynamically regulate HSD17B4 protein levels in PCa, we cultured LNCaP cells under hormone-deprived conditions for 24 h, followed by treating the cells with DHT at different concentrations for another 24 h, and found that 10 nM extracellular DHT dramatically decreased the protein level of HSD17B4 (Figure 4A). Further results indicated that DHT promoted HSD17B4 degradation in a time-dependent manner (Figure 4B). The degradation of HSD17B4 triggered by DHT also suggests a negative feedback mechanism. A previous study showed that K669 acetylation promotes HSD17B4 degradation through CMA [22]. We then examined the K669 acetylation level and found that 10 nM DHT increased the K669 acetylation level in both a time-dependent and dose-dependent manner (Figure 4A–4B). Our data demonstrate that K669 acetylation can promote the degradation of HSD17B4 in PCa cells. In order to verify which mechanism is responsible for HSD17B4 degradation, we treated LNCaP cells with the ubiquitin proteasome inhibitor MG132 and the lysosomal inhibitor chloroquine (CLQ). Then, we determined the levels of total HSD17B4 protein by western blotting. The results showed that CLQ, but not MG132, restored the HSD17B4 protein level, which was reduced by physiological signal treatment (Figure 4C–4D), indicating that the degradation of HSD17B4 is independent of the proteasome pathway. Thus, we speculated that the lysosome system may participate in the degradation of HSD17B4.

### The degradation of HSD17B4 requires CMA

**Figure 5:**
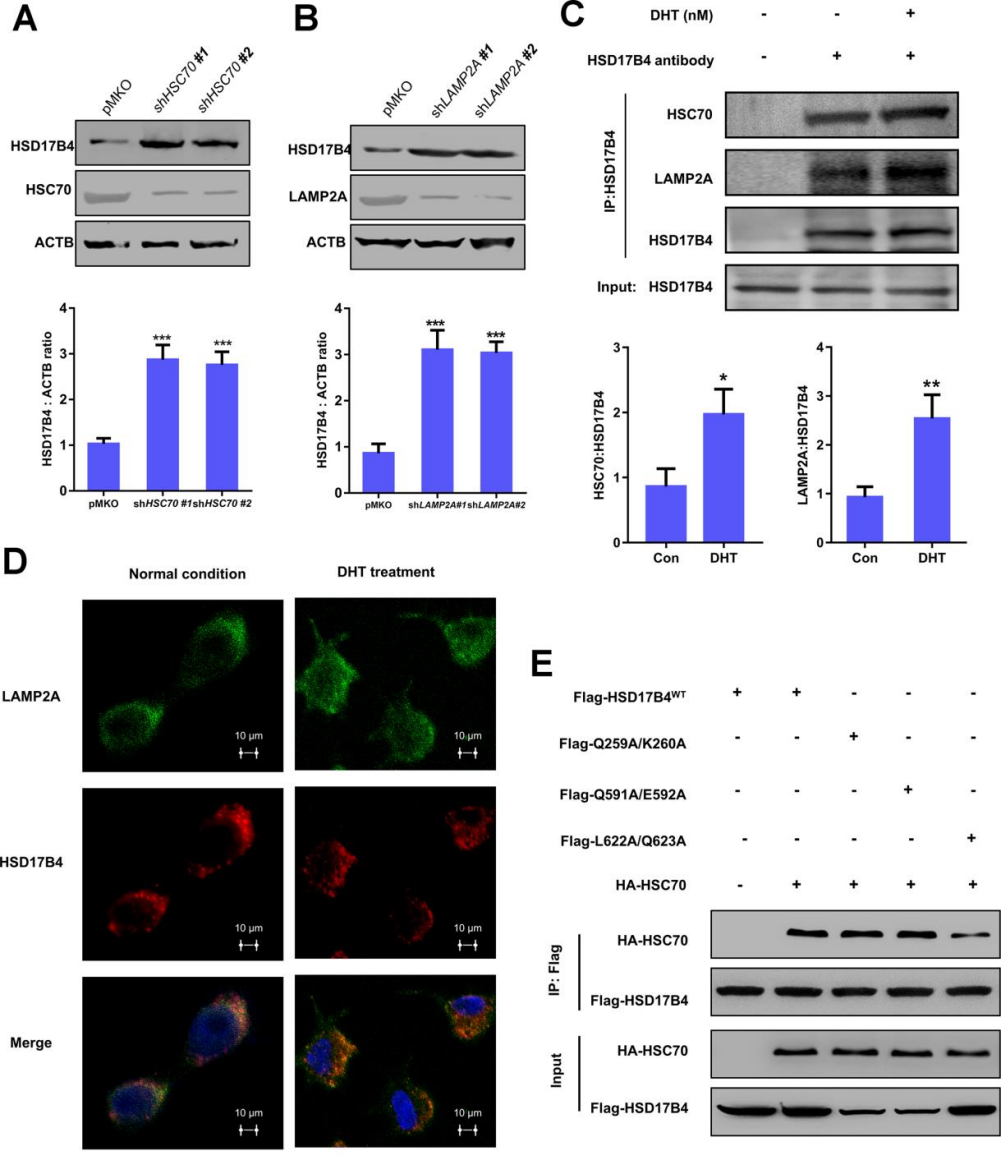
**The degradation of HSD17B4 requires CMA.** (**A**) *HSC70* knockdown leads to accumulation of HSD17B4. *HSC70* was transiently knocked down in LNCaP cells by shRNA. The knockdown efficiency and HSD17B4 protein level were determined by western blotting (upper panel). The HSD17B4 level was normalized against ACTB. ^**^denotes P < 0.01. Error bars represent ±SD of triplicate experiments (lower panel). (**B**) *LAMP2A* knockdown leads to accumulation of HSD17B4. LNCaP cells stably expressed *LAMP2A* shRNA. The levels of HSD17B4 and LAMP2A proteins were determined by western blotting (upper panel). The relative HSD17B4 protein compared with ACTB was quantified. ^**^denotes P < 0.01. Error bars represent ±SD of triplicate experiments (lower panel). (**C**) DHT promotes endogenous HSD17B4 binding with HSC70 and LAMP2A in LNCaP cells. LNCaP cells were cultured with or without DHT for 24 h before harvest. The interaction between endogenous HSD17B4 and HSC70 or LAMP2A was determined by coimmunoprecipitation and western blotting. (**D**) DHT enhances the interaction between HSD17B4 and LAMP2A. LNCaP cells were cultured with or without DHT for 24h as indicated and then paraformaldehyde fixed, blocked, and processed for double immunofluorescence with antibodies against LAMP2A (green) and HSD17B4 (red). Merged images of both channels are shown in the lower panel. Bar: 10 μm. (**E**) Identification of an HSC70 binding motif in HSD17B4. The binding between HSD17B4 mutants and HSC70 was analyzed by IP-western. Data are shown as the mean ± SD (n = 3) or typical photographs of one representative experiment. Similar results were obtained in three independent experiments. *p < 0.05, **p < 0.01, ***p <0.001.

There are three classic lysosomal autophagy mechanisms, namely, macroautophagy, microautophagy and CMA, that participate in the degradation of multiple proteins [23]. In the CMA pathway, the Heat Shock Protein Family A Member 8 (HSC70) carries target proteins to the lysosomal receptor LAMP2A. Then, LAMP2A translocates the target proteins into lysosomes for degradation [24]. To identify which mechanism is responsible for HSD17B4 degradation, we knocked down *HSC70* and *LAMP2A* in LNCaP cells and observed a significant increase in HSD17B4 protein (Figure 5A–5B). Moreover, extracellular DHT not only exclusively increased the interaction between HSC70 and HSD17B4 but also the interaction between HSD17B4 and LAMP2A (Figure 5C). Consistently, costaining of HSD17B4 and the lysosomal marker LAMP2A also indicates that DHT promotes the colocalization of HSD17B4 and LAMP2A (Figure 5D), consistent with a promoting role of DHT in HSD17B4 degradation through CMA. These data implied the involvement of CMA in HSD17B4 degradation.

As previously mentioned, substrates of CMA are recognized by the molecular chaperone HSC70. CMA targets various misfolded cytosolic proteins, especially those containing the KFERQ pentapeptide sequence [25]. The KFERQ motif usually consists of a critical glutamine (Q), followed by four amino acids consisting of a basic (R or K), an acidic (E or D), or a bulky hydrophobic residue (I, L, V, or F) [26]. However, the binding motif of HSD17B4 with HSC70 remains unclear. Based on this information, we found three potential HSC70 binding motifs: ^256^IVRQK^260^, ^586^QTKVQE^592^, and ^621^KLQSTFVFEE^630^. We first replaced Q259/K260, Q591/E592, and L622/Q623 with alanine. Next, we analyzed the binding between HSD17B4 and HSC70 by co-IP and western blotting to validate the key binding motif in HSD17B4. The results demonstrated that the mutation of L622A/Q623A significantly decreased the binding between HSD17B4 and HSC70 (Figure 5E). Taken together, our results support that HSD17B4 is degraded via the CMA pathway. The L622/Q623 residue of the KFERQ penta-peptide sequence may be the HSC70 binding motif in HSD17B4.

### SIRT3 and CREBBP co-regulate the acetylation level and protein level of HSD17B4

**Figure 6:**
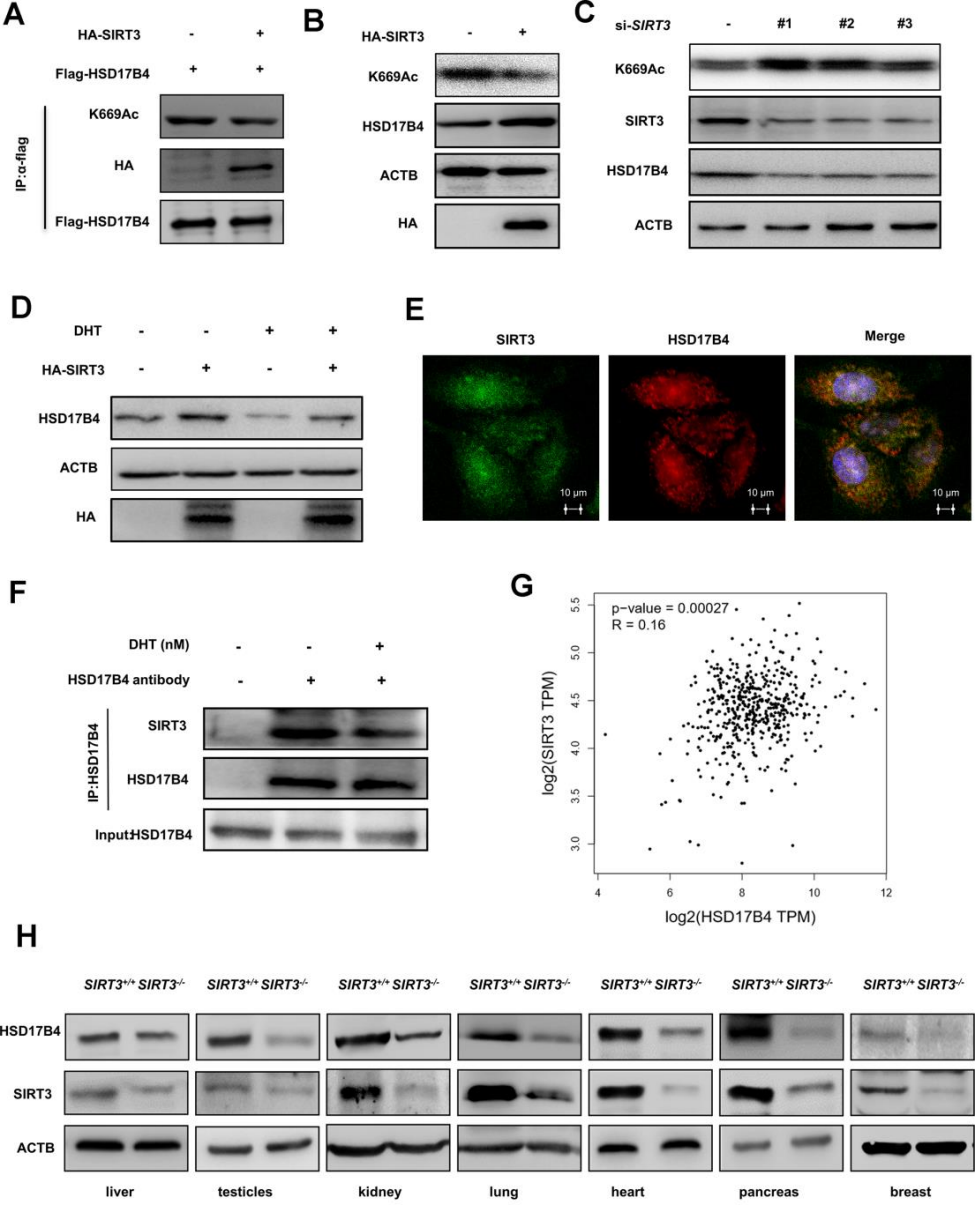
**SIRT3 decreases the K669 acetylation level and stabilizes the protein level of HSD17B4.** (**A**) SIRT3 overexpression decreases HSD17B4 acetylation. HA-tagged SIRT3 plasmid was cotransfected with FLAG-tagged HSD17B4 plasmid into LNCaP cells. Proteins were immunoprecipitated for western blotting. (**B**) SIRT3 overexpression causes accumulation of HSD17B4 protein. LNCaP cells were transfected with HA-SIRT3, and the protein levels of endogenous HSD17B4 were determined by western blotting. (**C**) SIRT3 knockdown decreases HSD17B4 protein. LNCaP cells were transfected with si*SIRT3* or control. Cells were harvested 48 h after transfection, and cell lysates were analyzed by western blotting. (**D**) SIRT3 can rescue the protein levels of HSD17B4 that are decreased by DHT. LNCaP cells were transfected with HA-SIRT3 and untreated or treated with DHT. HSD17B4 protein levels were determined by western blotting. (**E**) SIRT3 colocalizes with HSD17B4. Cultured LNCaP cells were paraformaldehyde fixed, blocked, and processed for double immunofluorescence with antibodies against SIRT3 (green) and HSD17B4 (red). Merged images of both channels are shown on the right. Bar: 10 μm. (**F**) DHT decreases endogenous HSD17B4 binding with SIRT3. LNCaP cells were cultured with or without DHT for 24 h before harvest. The interaction between endogenous HSD17B4 and SIRT3 was determined by co-IP and western blotting. (**G**) SIRT3 is positively correlated with HSD17B4 in PCa. The correlation between the mRNA expression levels of SIRT3 and HSD17B4 in patients with PCa was analyzed using the public web server GEPIA. (**H**) The expression of SIRT3 and HSD17B4 in the *SIRT3* knockout mouse model. Whole cell lysates were prepared from different mouse tissues, followed by western blotting analysis. Data are shown as the mean ± SD (n = 3) or typical photographs of one representative experiment. Similar results were obtained in three independent experiments. *p < 0.05, **p < 0.01, ***p <0.001.

Previous studies have suggested that SIRT3 is responsible for acetylation of HSD17B4 in breast cancer cells [22]. We transfected HA-tagged SIRT3 and FLAG-tagged HSD17B4 into LNCaP cells and determined the acetylation level of HSD17B4 by western blotting. As a result, HA-tagged SIRT3 significantly decreased the K669 acetylation level of HSD17B4 (Figure 6A). A previous study reported that acetylation promotes HSD17B4 degradation. Therefore, we investigated whether SIRT3 affected the protein level of HSD17B4 in PCa. We transfected HA-tagged SIRT3 into LNCaP cells and observed that SIRT3 overexpression caused a decrease in K669 acetylation but an accumulation of HSD17B4 protein (Figure 6B). Consistently, *SIRT3* knockdown increased K669 acetylation and reduced HSD17B4 protein levels at the same time (Figure 6C). Moreover, we treated LNCaP cells with or without 10 nM DHT and found that SIRT3 restored the protein level of HSD17B4, which emphasized the important role of SIRT3 in stabilizing HSD17B4 (Figure 6D). Confocal microscopy data indicated an interaction between SIRT3 and HSD17B4 (Figure 6E). However, DHT exclusively decreased the interaction between endogenous SIRT3 and HSD17B4 (Figure 6F). According to previous studies, SIRT3 mainly functions in the mitochondria, while HSD17B4 plays a major role in peroxisomes [27, 28]. We then analyzed the relationship between SIRT3 and HSD17B4 in PCa patients via the GEPIA (Gene Expression Profiling Interactive Analysis) web server and found that SIRT3 is positively correlated with HSD17B4 (Figure 6G). To better understand whether SIRT3 regulates HSD17B4 protein levels in vivo, pairs of normal mouse and *SIRT3* knockout mouse models were applied. Taking advantage of the mouse model, we collected different mouse tissues and detected endogenous HSD17B4 and SIRT3 protein levels. The results showed a positive correlation between SIRT3 and HSD17B4 (Figure 6H). Collectively, these data demonstrate that SIRT3 regulates HSD17B4 acetylation and blocks HSD17B4 degradation induced by DHT treatment.

**Figure 7:**
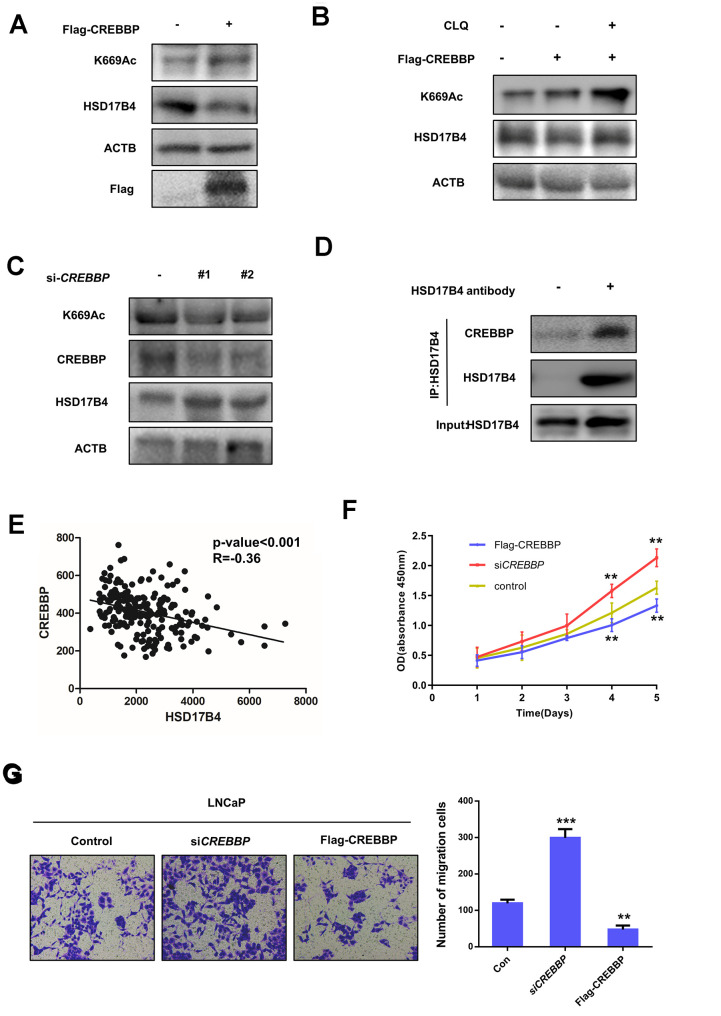
**CREBBP increases K669 acetylation levels and promotes the degradation of HSD17B4.** (**A**) CREBBP overexpression increases the K669 acetylation level but decreases the HSD17B4 protein level. LNCaP cells were transfected as indicated. Cell lysates were subjected to western blotting. The K669 acetylation level and HSD17B4 levels were normalized against ACTB. (**B**) CLQ blocks the decrease in HSD17B4 protein induced by CREBBP overexpression. FLAG-CREBBP was transfected into LNCaP cells with or without CLQ treatment and then subjected to western blotting. (**C**) The knockdown of *CREBBP* decreases HSD17B4 K669 acetylation while increasing its protein level. LNCaP cells were transfected with si*CREBBP* or control. The acetylation and protein levels of HSD17B4 and the protein level of CREBBP were determined by western blotting and were normalized against ACTB. (**D**) Endogenous HSD17B4 binds CREBBP in LNCaP cells. LNCaP cells were cultured and harvested until the cell density reached 90%. The interaction between endogenous HSD17B4 and CREBBP was determined by co-IP and western blotting. (**E**) CREBBP is negatively correlated with HSD17B4 in PCa. The correlation between the mRNA expression levels of CREBBP and HSD17B4 in patients with PCa was analyzed using the public dataset GSE70770. (**F**) *CREBBP* knockdown promotes LNCaP cell growth, while *CREBBP* overexpression inhibits cell growth. The CCK-8 assay showed that the proliferation rate of LNCaP cells was affected by *CREBBP* knockdown or overexpression. The data shown are representative of three independent experiments. (**G**) *CREBBP* knockdown promotes the migration of LNCaP cells, while *CREBBP* overexpression acts the opposite way. LNCaP cells were transfected with si*CREBBP* or Flag-CREBBP as indicated, followed by a migration assay in 24-well chambers without Matrigel. Quantitative analysis of cell migration in 24-well chambers was performed by ImageJ. Data are shown as the mean ± SD (n = 3) or typical photographs of one representative experiment. Similar results were obtained in three independent experiments. *p < 0.05, **p < 0.01, ***p <0.001.

In addition, we found that the overexpression of CREBBP in LNCaP cells obviously increased the HSD17B4 K669 acetylation level but decreased its protein level inversely (Figure 7A). This downregulation of HSD17B4 protein could be blocked by CLQ treatment (Figure 7B), indicating the positive role of CREBBP in K669 acetylation-induced CMA degradation of HSD17B4. Further experiments showed that CREBBP knockdown dramatically decreased K669 acetylation but increased HSD17B4 protein levels (Figure 7C). We observed a direct interaction between endogenous HSD17B4 and CREBBP in LNCaP cells (Figure 7D). Moreover, the analysis based on the GSE70770 database showed that CREBBP is negatively correlated with HSD17B4 (Figure 7E). We then performed a CCK-8 cell proliferation assay and found that the overexpression of CREBBP significantly decreased the proliferation rate of LNCaP cells, while the knockdown of *CREBBP* by siRNA promoted the proliferation rate (Figure 7F). Consistently, the overexpression of CREBBP inhibited the migrating capability of LNCaP cells, while the knockdown group showed an increased number of migration cells compared with that in the control group (Figure 7G). Taken together, our data demonstrate that CREBBP plays an important role in HSD17B4 degradation and may be the main acetyltransferase responsible for HSD17B4 acetylation.

### K669 acetylation is downregulated in human PCa tissues

**Figure 8:**
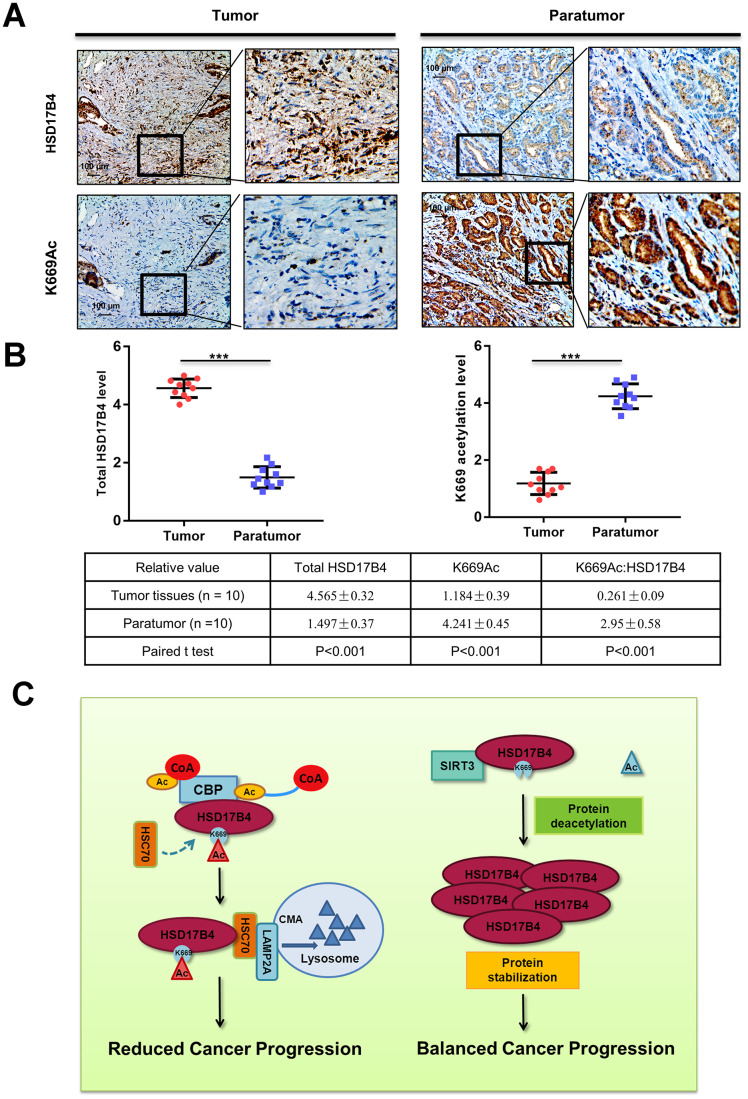
**K669 acetylation is downregulated in human PCa samples.** (**A**–**B**) Immunohistochemical staining of K669-acetylated and total HSD17B4 proteins in tumor and adjacent tissues. Examples are shown in (**A**), and the statistical analysis of all samples is shown in (**B**). Scale bars: 100 mm. The intensities of the K669-acetylated (left panel) and total (right panel) HSD17B4 protein were quantified, followed by statistical analysis. A total of 10 PCa tissues and 10 adjacent normal prostate tissues were analyzed. The mean value of multiple samples and the standard deviation are presented. (**C**) Working model. The increase in K669 acetylation promotes HSD17B4 degradation via CMA and then slows down the progression of PCa. CREBBP increases the HSD17B4 acetylation level and promotes its degradation, while SIRT3 can deacetylate K669 acetylation and stabilize its protein level. The accumulation of HSD17B4 can lead to consistent proliferation and migration of PCa cells. Data are shown as the mean ± SD (n = 3) or typical photographs of one representative experiment. Similar results were obtained in three independent experiments. *p < 0.05, **p < 0.01, ***p <0.001.

To substantiate the important role of K669 acetylation in PCa, we collected a total of 10 primary human PCa samples and 10 adjacent normal samples. Taking advantage of the anti-acetyl-HSD17B4 (K669) antibody, we performed immunohistochemistry in these PCa tissues and adjacent normal prostate tissues. Compared with the normal prostate tissues, the levels of total HSD17B4 were higher and the levels of relative K669-acetylated HSD17B4 were lower in the tumor tissues (Figure 8A). Statistical analyses of quantified images indicated that the differences in total HSD17B4 protein levels (P < 0.001), in K669-acetylated HSD17B4 (P < 0.001), and in the ratio of K669-acetylated HSD17B4 versus total HSD17B4 proteins (P < 0.001) between tumor and normal tissues are all significant (Figure 8B). These data further validate that K669 acetylation of HSD17B4 promotes its degradation in human PCa tissues.

## DISCUSSION

HSD17B4 transforms active estrogen and androgen into less active forms and in turn regulates multiple genes involved in fatty acid and cholesterol metabolism [29]. Counterintuitively, studies implicate an increase in *HSD17B4* expression related to poor prognosis for PCa. Investigations of the biological function of HSD17B4 may provide evidence for therapeutic target design or as a candidate biomarker. In PCa, HSD17B4 displays multiple enzymatic functions, including bile acid biosynthesis, fatty acid β-oxidation, hormone metabolism and transportation, indicating that a promoting role of HSD17B4 in oncogenes is through changing the cellular metabolism of PCa [30]. Our present study demonstrates that CREBBP and SIRT3 control the K669 acetylation level of HSD17B4 and subsequently regulate its degradation (Figure 8C). In this paper, we report that the expression of HSD17B4 was increased as PCa progressed. Upregulating HSD17B4 enhanced the malignant capacities of PCa cells, while HSD17B4 knockdown inhibited these capacities. Moreover, the stability was affected by CREBBP and SIRT3 via altering the acetylated modification of HSD17B4. Meanwhile, the HSD17B4 acetylation level was negatively related to different disease stages. Thus, it might serve as a diagnostic biomarker to predict PCa development.

CMA is an intracellular catabolic pathway that mediates the degradation of a selective subset of cytosolic proteins in lysosomes [31, 32] and allows cells to survive in difficult circumstances, such as nutrient deprivation, inflammation, and hypoxia [31–33]. We demonstrate that K669 acetylation promotes HSD17B4 degradation via CMA. A key step in CMA regulation is the interaction between the chaperone HSC70 and target proteins [34]. In CMA, the target proteins are translocated into the lysosome for degradation [32], and all the target proteins contain a special peptide sequence KFERQ that can be recognized by HSC70 [35, 36]. Here, we show that the L622/Q623 residue of the KFERQ penta-peptide sequence may be the main binding motif in HSD17B4. Further structural studies will be needed to obtain a precise understanding of how HSC70 recognizes the target HSD17B4 protein.

Androgen is a small steroid molecule that is modified and transformed by a number of sterol metabolic enzymes. Unbalanced androgen levels are believed to play vital roles in PCa progression [37–39]. Moreover, inhibiting androgen synthesis is a major clinical treatment for PCa. Both the different kinds and different concentration levels of androgens have different effects on the enzymatic pathways and downstream metabolites [40]. Thus, different kinds and concentrations of androgens as well as the corresponding steroid metabolic enzymes can become the targets of PCa therapy. The expression or activity of related metabolic enzymes in PCa cells is often altered, including gene deletion, amplification, mRNA alterable splicing and posttranslational modification [4, 41–43]. Therefore, the study of enzymes is important for elucidating the mechanism of drug resistance, discovering new therapeutic targets and designing new drugs. Upregulation of HSD17B4 in PCa is in part due to the overexpression regulated by altered gene transcription or mRNA stability [15]. Here, we found that the HSD17B4 acetylation level is low in PCa tissues when compared with adjacent tissues. Moreover, we propose that HSD17B4 acetylation modulated by the DHT concentration plays a crucial role in HSD17B4 stability control.

In conclusion, we demonstrate that K669 acetylation promotes HSD17B4 degradation via the CMA pathway in LNCaP cells. SIRT3 and CREBBP co-regulate the acetylation level of HSD17B4. SIRT3 decreases the K669 acetylation level and stabilizes the HSD17B4 protein level, while CREBBP has the opposite effect. Notably, the regulatory mechanism of HSD17B4 with its related interactors provides new insight into prostate cancer progression and indicates that androgen metabolic enzymes are potential biomarkers.

## MATERIALS AND METHODS

### Cell culture and treatment

Cells were all purchased from American Type Culture Collection and cultured as previously described [44]. LNCaP, VCaP, DU145 and PC3 cells were cultured in RPMI-1640 medium (HyClone, SH30234.01), containing 10% fetal bovine serum (FBS; Biological Industries, 04–001–1ACS) and 1% penicillin and streptomycin (HyClone, SV30010). RWPE-1 cells were cultured in keratinocyte growth medium supplemented with 5 ng/ml human recombinant epidermal growth factor and 0.05 mg/ml bovine pituitary extract (Invitrogen Life Technologies, CA, USA) in a humidified environment at 37°C and 5% CO_2_. For DHT-added treatment, LNCaP cells were first cultured in medium containing fetal bovine serum without hormones purchased from Biological Industries (04–201–1). Then, DHT (Sigma-Aldrich, 46573) at different concentrations was added to the medium to culture cells. Proteasomal/lysosomal inhibitor treatments were performed by adding chloroquine (Sigma-Aldrich, C6628; 200 mM) for 24 h, MG132 (Abmole BioScience, M1902; 10 mM) for 6 h, leupeptin (Selleckchem, S7380; 0.4 mM) for 24 h, and BAF (Selleckchem, S1413; 100 nM) for 8 h. All concentrations are final concentrations in the culture medium.

### Immunoprecipitation and western blotting

Western blotting was performed as previously mentioned [45, 46]. PCa cells were lysed as indicated in 0.3% Nonidet P40 (Sigma-Aldrich, 74388) buffer containing 150 mM NaCl, 50 mM Tris-HCl, pH 7.5, and complete protease inhibitor cocktail (Roche, 04693132001). Cell lysates were incubated for 3 h at 4°C with anti-FLAG M2 agarose (Sigma-Aldrich, A2220) after removal of debris by centrifuging at 4°C, 15,000 g for 15 min. The immunoprecipitants were bound with anti-FLAG M2 agarose beads and washed with lysis buffer 3 times by centrifuging at 400 g for 1 min. The beads were boiled and centrifuged at 4°C before loading on 10% SDS-PAGE gels and transferred onto nitrocellulose membrane (GE Healthcare, 10402495) for western blotting analysis. The following primary antibodies were commercially obtained: FLAG (Sigma-Aldrich, F3165; 1:10,000 working dilution), HA (Santa Cruz Biotechnology, 7392; 1:1,000 working dilution), ACTB (Sigma-Aldrich, A5441; 1:10,000 working dilution), HSC70 (Abcam, 19136; 1:1,000 working dilution), HSD17B4 (Abcam, 128565; 1:1,000 working dilution), SIRT3 (Abcam, 45067; 1:1,000 working dilution) and acetylated-lysine (Cell Signaling Technology, 9441; 1:1,000 working dilution). K669Ac was prepared commercially from immunized rabbits at Shanghai Genomic Inc. (1:1000 working dilution).

### Immunofluorescence staining

LNCaP cells were treated with DHT for 24 h. Then, cells were fixed in 4% paraformaldehyde (Wuhan Goodbio technology, G1101), permeabilized with 0.2% Triton X-100 (Sigma-Aldrich, T8787), blocked with 5% bovine serum albumin (Amresco, 0332) in phosphate buffered saline (Sigma-Aldrich, P5368), and incubated with the indicated primary antibodies: LAMP2A (1:500 working dilution), HSD17B4 (1:500 working dilution), SIRT3 (1:500 working dilution), and Catalases (Abcam, 16731; 1:500 working dilution). Detection was performed with corresponding fluorescent-conjugated secondary antibodies. Confocal fluorescence images were randomly obtained with a confocal microscope.

### Knockdown and overexpressing stable cell lines

pMKO-sh*VEC* or pMKO-sh*HSD17B4* was constructed as a short hairpin RNA vector that employed 3 effective sequences targeting the 3’-untranslated region as follows: 5’-AGGGCACACTACACTATTAAT-3’; 5’-GCCCAAGTCCTGTTTCCTTAG-3’; and 5’-GCTCTGCTTGTTCGTGTGTGT-3’. GFP and FLAG-tagged human HSD17B4 were cloned into the retroviral vector pQCXIH. All pMKO-sh*VEC*, pMKO-sh*HSD17B4*, pQCXIH-GFP and pQCXIH-HSD17B4 vectors were transfected into HEK293T cells, together with vectors expressing gag and vsvg genes (from vesicular stomatitis virus G) to produce retrovirus. Then, PCa (LNCaP, DU145 and PC3) cells were infected with pQCXIH-HSD17B4, pQCXIH-GFP, pMKO-sh*HSD17B4* or pMKO-sh*VEC* retroviral supernatants. Polybrene (8 mg/ml) was added to PCa cells to increase the infection efficiency. The *HSD17B4* knockdown cell pool was selected with puromycin (Amresco, J593; 5 mg/ml final concentration), while the HSD17B4 overexpression cell pool was screened using hygromycin (Amresco, K547; 350 mg/ml final concentration) for at least 2 weeks. The knockdown or overexpression efficiency of positive clones was determined by western blotting.

### Cell migration and wound healing assay

PCa cells (2×10^5^) stably expressing HSD17B4 were suspended in 100 μl of serum-free medium. For the upper embedded culture chambers, 200 μl of cell suspensions was added. For the bottom culture chamber, 600 μl of RPMI-1640 medium with 10% FBS was added. Three repeated wells were set for each group. The experiment was conducted in 5% CO_2_ at 37°C for 48 h. The liquid in the chambers was ejected, and cells on the internal surface of the chamber bottom were wiped with a cotton swab, fixed with paraformaldehyde, stained with crystal violet, and rinsed with phosphate buffered saline. Five random views were chosen for each culture well under the light microscope, and the number of cells in each view was counted. For the wound healing assay, LNCaP cells (2×10^5^) stably expressing HSD17B4 were seeded in 6-well plates. When confluency was reached, the cell layer was scraped with a 10-μl pipette tip. Cell migration was observed by microscopy 24 h later. All biochemical assays were performed in three independent experiments.

### Colony formation assay

PCa cells were plated in 6-well plates (1.0 × 10^3^ cells per well), cultured for 7 days, fixed with paraformaldehyde for 10 min, and stained with 1% crystal violet for 5 min before counting the number of colonies.

### CCK-8 assay

Cell proliferation was measured using the Cell Counting Kit 8 (CCK-8; Dojindo, Kumamoto, Japan). Briefly, PCa cells were seeded in 96-well plates in triplicate at an initial density of 3 × 10^3^ cells/well. Then, 10 μl of CCK-8 assay solution was added to each well and incubated for 2 h. The absorbance was measured at 450 nm for each well using a multi-well spectrophotometer.

### RNAseq data processing and visualization

The RNA sequence data of 498 prostate cancer patients were retrieved from the TCGA data portal. Data were downloaded from the TCGA data coordination center. This dataset shows the gene-level transcription estimates as the log2(x+1) transformed RSEM normalized count. Genes are mapped onto the human genome coordinates using the UCSC Xena HUGO probeMap (see ID/Gene mapping as below for details). This study is in accordance with the publication guidelines provided by TCGA (https://cancergenome.nih.gov/publications/publicationguidelines).

### SIRT3 knockout mouse model

The *SIRT3^-/-^* mice were purchased from the Jackson Laboratory (Stock No: 012755). The procedures related to animal studies were approved by the Ethics Committee of XiangYa Hospital, Central South University.

### PCa samples and immunohistochemistry

PCa samples were acquired from XiangYa Hospital of Central South University. A physician obtained informed consent from the patients. The procedures related to human subjects were approved by the Ethics Committee of XiangYa Hospital, Central South University. Immunohistochemistry (IHC) was performed as previously described [47, 48]. To quantify the IHC result of positive staining, 5 random areas in each tissue sample were microscopically examined and analyzed by an experienced pathologist. The average staining score was calculated by dividing the positive areas by the total areas. The data obtained are expressed as the mean values ± SD.

### Statistical analysis

Two-tailed Student’s *t*-tests were used for all comparisons. All values included in the figures represent the mean values ± SD. Error bars represent ±SD for triplicate experiments. The statistical significance is indicated as asterisks (*). A 2-sided P value of < 0.05 was considered statistically significant (*P < 0.05, **P < 0.01, ***P < 0.001).

## Supplementary Material

Supplementary Figures

Supplementary Table 1

Supplementary Table 2

## References

[1] Siegel R, Naishadham D, Jemal A. Cancer statistics, 2013. CA Cancer J Clin. 2013; 63:11–30. 10.3322/caac.21166 23335087

[2] Penning TM, Byrns MC. Steroid hormone transforming aldo-keto reductases and cancer. Ann N Y Acad Sci. 2009; 1155:33–42. 10.1111/j.1749-6632.2009.03700.x 19250190 PMC3038333

[3] Penning TM. Mechanisms of drug resistance that target the androgen axis in castration resistant prostate cancer (CRPC). J Steroid Biochem Mol Biol. 2015; 153:105–13. 10.1016/j.jsbmb.2015.05.010 26032458 PMC4568163

[4] Montgomery RB, Mostaghel EA, Vessella R, Hess DL, Kalhorn TF, Higano CS, True LD, Nelson PS. Maintenance of intratumoral androgens in metastatic prostate cancer: a mechanism for castration-resistant tumor growth. Cancer Res. 2008; 68:4447–54. 10.1158/0008-5472.CAN-08-0249 18519708 PMC2536685

[5] Chang KH, Li R, Kuri B, Lotan Y, Roehrborn CG, Liu J, Vessella R, Nelson PS, Kapur P, Guo X, Mirzaei H, Auchus RJ, Sharifi N. A gain-of-function mutation in DHT synthesis in castration-resistant prostate cancer. Cell. 2013; 154:1074–84. 10.1016/j.cell.2013.07.029 23993097 PMC3931012

[6] Adeniji AO, Chen M, Penning TM. AKR1C3 as a target in castrate resistant prostate cancer. J Steroid Biochem Mol Biol. 2013; 137:136–49. 10.1016/j.jsbmb.2013.05.012 23748150 PMC3805777

[7] Ko HK, Berk M, Chung YM, Willard B, Bareja R, Rubin M, Sboner A, Sharifi N. Loss of an androgen-inactivating and isoform-specific HSD17B4 splice form enables emergence of castration-resistant prostate cancer. Cell Rep. 2018; 22:809–19. 10.1016/j.celrep.2017.12.081 29346776 PMC5798464

[8] Castagnetta LA, Carruba G, Traina A, Granata OM, Markus M, Pavone-Macaluso M, Blomquist CH, Adamski J. Expression of different 17beta-hydroxysteroid dehydrogenase types and their activities in human prostate cancer cells. Endocrinology. 1997; 138:4876–82. 10.1210/endo.138.11.5497 9348218

[9] Lu X, Ma P, Shi Y, Yao M, Hou L, Zhang P, Jiang L. NF-κB increased expression of 17β-hydroxysteroid dehydrogenase 4 promotes HepG2 proliferation via inactivating estradiol. Mol Cell Endocrinol. 2015; 401:1–11. 10.1016/j.mce.2014.11.016 25448063

[10] Rasiah KK, Gardiner-Garden M, Padilla EJ, Möller G, Kench JG, Alles MC, Eggleton SA, Stricker PD, Adamski J, Sutherland RL, Henshall SM, Hayes VM. HSD17B4 overexpression, an independent biomarker of poor patient outcome in prostate cancer. Mol Cell Endocrinol. 2009; 301:89–96. 10.1016/j.mce.2008.11.021 19100308

[11] English MA, Kane KF, Cruickshank N, Langman MJ, Stewart PM, Hewison M. Loss of estrogen inactivation in colonic cancer. J Clin Endocrinol Metab. 1999; 84:2080–85. 10.1210/jcem.84.6.5772 10372714

[12] Nagayoshi Y, Ohba T, Yamamoto H, Miyahara Y, Tashiro H, Katabuchi H, Okamura H. Characterization of 17beta-hydroxysteroid dehydrogenase type 4 in human ovarian surface epithelial cells. Mol Hum Reprod. 2005; 11:615–21. 10.1093/molehr/gah215 16219629

[13] Lin R, Tao R, Gao X, Li T, Zhou X, Guan KL, Xiong Y, Lei QY. Acetylation stabilizes ATP-citrate lyase to promote lipid biosynthesis and tumor growth. Mol Cell. 2013; 51:506–18. 10.1016/j.molcel.2013.07.002 23932781 PMC4180208

[14] Yang HB, Xu YY, Zhao XN, Zou SW, Zhang Y, Zhang M, Li JT, Ren F, Wang LY, Lei QY. Acetylation of MAT IIα represses tumour cell growth and is decreased in human hepatocellular cancer. Nat Commun. 2015; 6:6973. 10.1038/ncomms7973 25925782 PMC4421817

[15] Lv L, Li D, Zhao D, Lin R, Chu Y, Zhang H, Zha Z, Liu Y, Li Z, Xu Y, Wang G, Huang Y, Xiong Y, et al. Acetylation targets the M2 isoform of pyruvate kinase for degradation through chaperone-mediated autophagy and promotes tumor growth. Mol Cell. 2011; 42:719–30. 10.1016/j.molcel.2011.04.025 21700219 PMC4879880

[16] Zhao D, Zou SW, Liu Y, Zhou X, Mo Y, Wang P, Xu YH, Dong B, Xiong Y, Lei QY, Guan KL. Lysine-5 acetylation negatively regulates lactate dehydrogenase a and is decreased in pancreatic cancer. Cancer Cell. 2013; 23:464–76. 10.1016/j.ccr.2013.02.005 23523103 PMC3885615

[17] Wang JH, Tuohimaa P. Regulation of 17beta-hydroxysteroid dehydrogenase type 2, type 4 and type 5 by calcitriol, LXR agonist and 5alpha-dihydrotestosterone in human prostate cancer cells. J Steroid Biochem Mol Biol. 2007; 107:100–05. 10.1016/j.jsbmb.2007.02.009 17627817

[18] Gerdes J, Schwab U, Lemke H, Stein H. Production of a mouse monoclonal antibody reactive with a human nuclear antigen associated with cell proliferation. Int J Cancer. 1983; 31:13–20. 10.1002/ijc.2910310104 6339421

[19] Weigel MT, Dowsett M. Current and emerging biomarkers in breast cancer: prognosis and prediction. Endocr Relat Cancer. 2010; 17:R245–62. 10.1677/ERC-10-0136 20647302

[20] Jones RL, Salter J, A’Hern R, Nerurkar A, Parton M, Reis-Filho JS, Smith IE, Dowsett M. The prognostic significance of Ki67 before and after neoadjuvant chemotherapy in breast cancer. Breast Cancer Res Treat. 2009; 116:53–68. 10.1007/s10549-008-0081-7 18592370

[21] Yerushalmi R, Woods R, Ravdin PM, Hayes MM, Gelmon KA. Ki67 in breast cancer: prognostic and predictive potential. Lancet Oncol. 2010; 11:174–83. 10.1016/S1470-2045(09)70262-1 20152769

[22] Zhang Y, Xu YY, Yao CB, Li JT, Zhao XN, Yang HB, Zhang M, Yin M, Chen J, Lei QY. Acetylation targets HSD17B4 for degradation via the CMA pathway in response to estrone. Autophagy. 2017; 13:538–53. 10.1080/15548627.2016.1268302 28296597 PMC5361611

[23] Mizushima N, Levine B, Cuervo AM, Klionsky DJ. Autophagy fights disease through cellular self-digestion. Nature. 2008; 451:1069–75. 10.1038/nature06639 18305538 PMC2670399

[24] Mizushima N, Komatsu M. Autophagy: renovation of cells and tissues. Cell. 2011; 147:728–41. 10.1016/j.cell.2011.10.026 22078875

[25] Agarraberes FA, Terlecky SR, Dice JF. An intralysosomal hsp70 is required for a selective pathway of lysosomal protein degradation. J Cell Biol. 1997; 137:825–34. 10.1083/jcb.137.4.825 9151685 PMC2139836

[26] Dice JF. Peptide sequences that target cytosolic proteins for lysosomal proteolysis. Trends Biochem Sci. 1990; 15:305–09. 10.1016/0968-0004(90)90019-8 2204156

[27] Onyango P, Celic I, McCaffery JM, Boeke JD, Feinberg AP. SIRT3, a human SIR2 homologue, is an NAD-dependent deacetylase localized to mitochondria. Proc Natl Acad Sci USA. 2002; 99:13653–58. 10.1073/pnas.222538099 12374852 PMC129731

[28] Lieber DS, Hershman SG, Slate NG, Calvo SE, Sims KB, Schmahmann JD, Mootha VK. Next generation sequencing with copy number variant detection expands the phenotypic spectrum of HSD17B4-deficiency. BMC Med Genet. 2014; 15:30. 10.1186/1471-2350-15-30 24602372 PMC4015298

[29] Swinnen JV, Ulrix W, Heyns W, Verhoeven G. Coordinate regulation of lipogenic gene expression by androgens: evidence for a cascade mechanism involving sterol regulatory element binding proteins. Proc Natl Acad Sci USA. 1997; 94:12975–80. 10.1073/pnas.94.24.12975 9371785 PMC24248

[30] Zha S, Ferdinandusse S, Hicks JL, Denis S, Dunn TA, Wanders RJ, Luo J, De Marzo AM, Isaacs WB. Peroxisomal branched chain fatty acid beta-oxidation pathway is upregulated in prostate cancer. Prostate. 2005; 63:316–23. 10.1002/pros.20177 15599942

[31] Dice JF. Chaperone-mediated autophagy. Autophagy. 2007; 3:295–99. 10.4161/auto.4144 17404494

[32] Cuervo AM. Chaperone-mediated autophagy: selectivity pays off. Trends Endocrinol Metab. 2010; 21:142–50. 10.1016/j.tem.2009.10.003 19857975 PMC2831144

[33] Saha T. LAMP2A overexpression in breast tumors promotes cancer cell survival via chaperone-mediated autophagy. Autophagy. 2012; 8:1643–56. 10.4161/auto.21654 22874552 PMC3494593

[34] Stricher F, Macri C, Ruff M, Muller S. HSPA8/HSC70 chaperone protein: structure, function, and chemical targeting. Autophagy. 2013; 9:1937–54. 10.4161/auto.26448 24121476

[35] Chiang HL, Terlecky SR, Plant CP, Dice JF. A role for a 70-kilodalton heat shock protein in lysosomal degradation of intracellular proteins. Science. 1989; 246:382–85. 10.1126/science.2799391 2799391

[36] Kaushik S, Cuervo AM. Chaperone-mediated autophagy: a unique way to enter the lysosome world. Trends Cell Biol. 2012; 22:407–17. 10.1016/j.tcb.2012.05.006 22748206 PMC3408550

[37] Attard G, Cooper CS, de Bono JS. Steroid hormone receptors in prostate cancer: a hard habit to break? Cancer Cell. 2009; 16:458–62. 10.1016/j.ccr.2009.11.006 19962664

[38] Mostaghel EA, Montgomery B, Nelson PS. Castration-resistant prostate cancer: targeting androgen metabolic pathways in recurrent disease. Urol Oncol. 2009; 27:251–57. 10.1016/j.urolonc.2009.03.016 19414113 PMC2705999

[39] Green SM, Mostaghel EA, Nelson PS. Androgen action and metabolism in prostate cancer. Mol Cell Endocrinol. 2012; 360:3–13. 10.1016/j.mce.2011.09.046 22453214 PMC4124858

[40] Ishizaki F, Nishiyama T, Kawasaki T, Miyashiro Y, Hara N, Takizawa I, Naito M, Takahashi K. Androgen deprivation promotes intratumoral synthesis of dihydrotestosterone from androgen metabolites in prostate cancer. Sci Rep. 2013; 3:1528. 10.1038/srep01528 23524847 PMC3607121

[41] Li Z, Alyamani M, Li J, Rogacki K, Abazeed M, Upadhyay SK, Balk SP, Taplin ME, Auchus RJ, Sharifi N. Redirecting abiraterone metabolism to fine-tune prostate cancer anti-androgen therapy. Nature. 2016; 533:547–51. 10.1038/nature17954 27225130 PMC5111629

[42] Li J, Alyamani M, Zhang A, Chang KH, Berk M, Li Z, Zhu Z, Petro M, Magi-Galluzzi C, Taplin ME, Garcia JA, Courtney K, Klein EA, Sharifi N. Aberrant corticosteroid metabolism in tumor cells enables GR takeover in enzalutamide resistant prostate cancer. Elife. 2017; 6:e20183. 10.7554/eLife.20183 28191869 PMC5305204

[43] Hearn JW, AbuAli G, Reichard CA, Reddy CA, Magi-Galluzzi C, Chang KH, Carlson R, Rangel L, Reagan K, Davis BJ, Karnes RJ, Kohli M, Tindall D, et al. HSD3B1 and resistance to androgen-deprivation therapy in prostate cancer: a retrospective, multicohort study. Lancet Oncol. 2016; 17:1435–44. 10.1016/S1470-2045(16)30227-3 27575027 PMC5135009

[44] Liu T, Wu HJ, Liang Y, Liang XJ, Huang HC, Zhao YZ, Liao QC, Chen YQ, Leng AM, Yuan WJ, Zhang GY, Peng J, Chen YH. Tumor-specific expression of shVEGF and suicide gene as a novel strategy for esophageal cancer therapy. World J Gastroenterol. 2016; 22:5342–52. 10.3748/wjg.v22.i23.5342 27340350 PMC4910655

[45] Li DJ, Deng G, Xiao ZQ, Yao HX, Li C, Peng F, Li MY, Zhang PF, Chen YH, Chen ZC. Identificating 14-3-3 sigma as a lymph node metastasis-related protein in human lung squamous carcinoma. Cancer Lett. 2009; 279:65–73. 10.1016/j.canlet.2009.01.028 19231067

[46] Li MX, Xiao ZQ, Chen YH, Peng F, Li C, Zhang PF, Li MY, Li F, Duan CJ, Li DJ, Yao HX, Chen ZC. Proteomic analysis of the stroma-related proteins in nasopharyngeal carcinoma and normal nasopharyngeal epithelial tissues. Med Oncol. 2010; 27:134–44. 10.1007/s12032-009-9184-1 19242827

[47] Mu Y, Chen Y, Zhang G, Zhan X, Li Y, Liu T, Li G, Li M, Xiao Z, Gong X, Chen Z. Identification of stromal differentially expressed proteins in the colon carcinoma by quantitative proteomics. Electrophoresis. 2013; 34:1679–92. 10.1002/elps.201200596 23737015

[48] Liu YF, Chen YH, Li MY, Zhang PF, Peng F, Li GQ, Xiao ZQ, Chen ZC. Quantitative proteomic analysis identifying three annexins as lymph node metastasis-related proteins in lung adenocarcinoma. Med Oncol. 2012; 29:174–84. 10.1007/s12032-010-9761-3 21132403

