# Supplementary material for: Acetylation-mediated degradation of HSD17B4 regulates the progression of prostate cancer
Source: Aging (Albany NY). 2020 Jul 17;12(14):14699–717. doi: 10.18632/aging.103530 (PMC7425433; doi:10.18632/aging.103530)
Supplement: Supplementary Figures [file aging-12-103530-s003..pdf]

SUPPLEMENTARY FIGURES

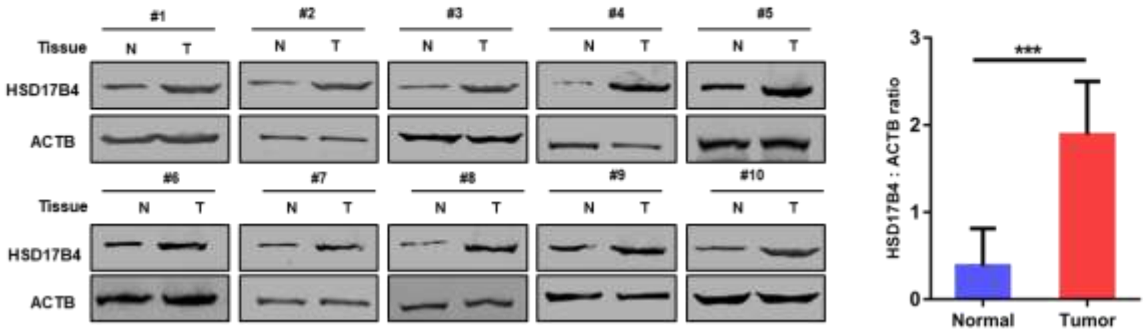

Supplementary Figure 1. Expression of HSD17B4 is increased as PCa develops. HSD17B4 is overexpressed in PCa tissues compared to expression in adjacent tissues. Human PCa samples each paired with cancerous tissue (designated as T) and adjacent normal tissue (designated as N) were lysed and directly subjected to western blotting. Ten pairs of samples clearly exhibited HSD17B4 overexpression in PCa tissues. The relative intensity of HSD17B4 and ACTB was determined using the ImageJ program. ***denotes P<0.001.

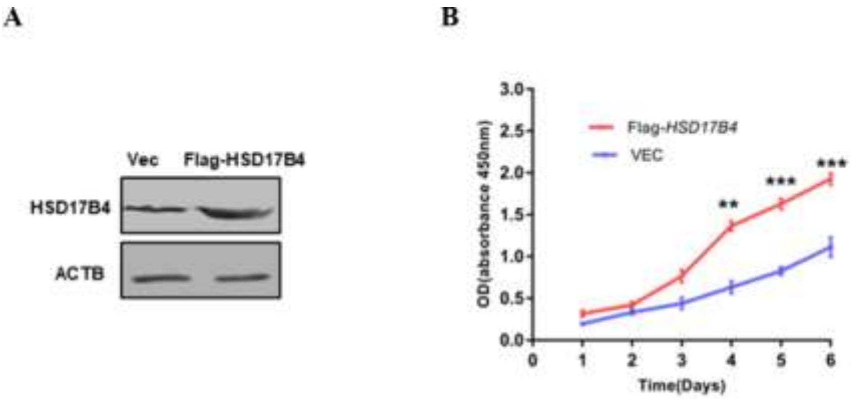

Supplementary Figure 2. HSD17B4 promotes the proliferation of PCa cells. (**A**) Verification of DU145 stable cell lines. The transfection efficiencies of Flag-HSD17B4 were determined by western blotting. (**B**) HSD17B4 overexpression promoted cell growth. The CCK-8 assay showed that the growth of DU145 cells characterized in (**A**) was affected by HSD17B4 overexpression. The data shown are representative of three independent experiments.
